# Supplementary material for: Whole-Exome Sequencing Identifies Homozygote Nonsense Variants in LMOD2 Gene Causing Infantile Dilated Cardiomyopathy
Source: Cells. 2023 May 23;12(11):1455. doi: 10.3390/cells12111455 (PMC10252268; doi:10.3390/cells12111455)
Supplement: Supplementary file 1 [file cells-12-01455-s001.zip › supple.pdf]

A

```
   1 agttgttgac cagcctgcca cttgcctccc tgcctgcttc tggccgcctt gaatgcctgg tccttcaagc tccttctggg tctgacaaag cagggaccat gtctaccttt ggctaccgaa
 121 gaggactcag taaatacgaa tccatcgacg aggatgaact cctcgcctcc ctgtcagccg aggagctgaa ggagctagag agagagttgg aagacattga acctgaccgc aaccttcccg
 241 tggggctaag gcaaaagagc ctgacagaga aaacccccac agggacattc agcagagagg cactgatggc ctattgggaa aaggagtccc aaaaactctt ggagaaggag aggctggggg
 361 aatgtggaaa ggttgcagaa gacaaagagg aaagtgaaga agagcttatc tttactgaaa gtaacagtga ggtttctgag gaagtgtata cagaggagga ggaggaggag tcccaggagg
 481 aagaggagga agaagacagt gacgaagagg aaagaacaat tgaaactgca aaagggatta atggaactgt aaattatgat agtgtcaatt ctgacaactc taagccaaag atatttaaaa
 601 gtcaaataga gaacataaat ttgaccaatg gcagcaatgg gaggaacaca gagtccccag ctgccattca cccttgtgga aatcctacag tgattgagga cgctttggac aagattaaaa
 721 gcaatgaccc tgacaccaca gaagtcaatt tgaacaacat tgagaacatc acaacacaga cccttacccg ctttgctgaa gccctcaagg acaacactgt ggtgaagacg ttcagtctgg
 841 ccaacacgca tgccgacgac agtgcagcca tggccattgc agagatgctc aaagtcaatg agcacatcac caacgtaaac gtcgagtcca acttcataac gggaaagggg atcctggcca
 961 tcatgagagc tctccagcac aacacggtgc tcacggagct gcgtttccat aaccagaggc acatcatggg cagccaggtg gaaatggaga ttgtcaagct gctgaaggag aacacgacgc
1081 tgctgaggct gggataccat tttgaactcc caggaccaag aatgagcatg acgagcattt tgacaagaaa tatggataaa cagaggcaaa aacgtttgca ggagcaaaaa cagcaggagg
1201 gatacgatgg aggacccaat cttaggacca aagtctggca aagaggaaca cctagctctt caccttatgt atctcccagg cactcaccct ggtcatcccc aaaactcccc aaaaaagtcc
1321 agactgtgag gagccgtcct ctgtctcctg tggccacacc tcctcctcct cccccctcctc ctcctcctcc ccctccttct tcccaaaggc tgccaccacc tcctcctcct cccccctcctc
1441 cactcccaga gaaaaagctc attaccagaa acattgcaga agtcatcaaa caacaggaga gtgcccaacg ggcattacaa aatggacaaa aaaagaaaaa agggaaaaag gtcaagaaac
1561 agccaaacag tattctaaag gaaataaaaa attctctgag gtcagtgcaa gagaagaaaa tggaagacag ttcccgacct tctaccccac agagatcagc tcatgagaat ctcatggaag
1681 caattcgggg aagcagcata aaacagctaa agcgggtgga agttccagaa gccctgcgat aaaaacatga tctttagaag aggatgcaga actgttcagt ggtattacat gaaatgcatt
1801 gtgagatgtt tctaaaatac cttcttcaat tcaaaatgat ccctgacttt aaaaataatc tcacccatta attccaaaga gaatcttaag aaacaatcag catgtttctt ctgtaaatat
1921 gaaaataaat ttctttttta tgtcgtgaga tttgtattgg caagaagcag ttaatttaaa gatgctcttc ctatctgtgg atgtgttggt aactccgagt tgtaatgagt tcatgaaatg
2041 tgctgttatt tttgtaatct caataaatgt ggattgaagt tttttccctt tttttaaagc caaactaata tttttctgtg acttgataca tctgtcagat ttttgtaatc tcgataaatg
2161 tgtattgaag tttttttccct ttttttaaaa agccaaacta atatttttct gtgagttaat acatctgtca ggtgtgtatg taacattact ggacattaaa aaaaaatatt acattctcac
2281 ccaaaaa
```

B

```
  1 mstfgyrrgl skyesidede llaslsaeel kelereledi epdrnlpvgl rqksltektp
 61 tgtfsrealm aywekesqkl lekerlgecg kvaedkeese eeliftesns evseevytee
121 eeeesqeeee eedsdeeert ietakgingt vnydsvnsdn skpkifksqi eninltngsn
181 grntespaai hpcgnptvie daldkiksnd pdttevnlnn ienittqtlt rfaealkdnt
241 vvktfslant haddsaamai aemlkvnehi tnvnvesnfi tgkgilaimr alqhntvlte
301 lrfhnqrhim gsqvemeivk llkenttllr lgyhfelpgp rmsmtsiltr nmdkqrqkrl
361 qeqkqqegyd ggpnlrtkvw qrgtpssspy vsprhspwss pklpkkvqtv rsrplspvat
421 pppppppppp pppssqrlpp pppppppplp ekklitrnia evikqqesaq ralqngqkkk
481 kgkkvkkqpn silkeiknsl rsvqekkmed ssrpstpqrs ahenlmeair gssikqlkrv
541 evpealr
```

Figure S1. ***LMOD2*** **Transcript and Amino Acid Sequences. A.** *LMOD2* transcript sequence**.** Opening (atg) and stop (taa) codons as well as splicing boundaries are underscored. Affected codons in the published reports are bolded in black. **B.** LMOD2 amino acid sequence and structural domains. Colored amino acids are those of the LMOD2 isoform 1~547 corresponding to the boxes on the domain diagram shown in **Figure 5. C.** with corresponding colors. The underscored amino acids are at the splicing boundaries. The bolded amino acids are the first affected amino acid based directly or indirectly on the sequencing results of reported cases.

Table S1. Biochemical testing and infection work up for infantile heart failure.

Differential Etiology	Test Parameters	Test Interpretation
Hypothyroidism	TSH<br>Free T4	Negative
Immune hemolytic anemia	Isotiter antibody screen	Negative
Congenital syphilis	RPR	Negative
SARS-CoV-19 infection	PCR/TMA	Negative
Viral hepatitis	HAV, HBV, HCV-PCR	Negative
HIV infection	HIV-1 and HIV-2 Ag/Ab screen 4th generation	Negative
HSV Infection	HSV-1 and HSV-2 DNA (PCR)	Negative
EBV infection	EBV DNA (PCR)	Negative
Rapid Respiratory Panel	Adenovirus, influenza A/B, human metapneumovirus, human rhinovirus/enterovirus, seasonal coronavirus, RSV A/B	Negative
CMV Infection	CMV DNA (PCR)	Negative
Para Influenza Infection	Para influenza Type 1,2,3,4 DNA (PCR)	Positive (Type 4)<br>Negative (Type 1, 2, 3)

PCR, polymerase chain reaction; TMA, transcription-mediated amplification; TSH, thyroid-stimulating hormone; HIV, human immunodeficiency virus; HSV, herpes simplex virus; EBV, Epstein-Barr virus; CMV, cytomegalic virus.

**Table S2.** Comprehensive Cardiomyopathy Panel*			
ABCC9	*DSG2*	*MAP2K1*	*RIT1*
ACADVL	*DSP*	*MAP2K2*	*RYR2*
ACTC1	*ELAC2*	*MRAS*	*SCN5A*
ACTN2	*EMD*	*MTO1*	*SDHA*
AGL	*EYA4*	*MYBPC3*	*SGCD*
ALMS1	*FHL1*	*MYH7*	*SHOC2*
ALPK3	*FKRP*	*MYL2*	*SLC22A5*
BAG3	*FKTN*	*MYL3*	*SOS1*
BRAF	*FLNC*	*MYL4*	*SOS2*
CACNA1C	*GAA*	*MYLK3*	*SPRED1*
CACNA1D	*GATA4*	*NF1*	*TAZ*
CALM1	*GATA5*	*NKX2-5*	*TBX20*
CALM2	*GJA5*	*NRAS*	*TCAP*
CALM3	*GLA*	*PCCA*	*TMEM43*
CASQ2	*HCN4*	*PCCB*	*TMEM70*
CBL	*HRAS*	*PKP2*	*TNNC1*
CDH2	*JUP*	*PLN*	*TNNI3*
CPT2	*KCNE1*	*PPA2*	*TNNI3K*
CRYAB	*KCNH2*	*PPCS*	*TNNT2*
CSRP3	*KCNJ2*	*PPP1CB*	*TPM1*
DES	*KCNQ1*	*PRKAG2*	*TRDN*
DMD	*KRAS*	*PTPN11*	*TRPM4*
DNAJC19	*LAMP2*	*RAF1*	*TTN*
DOLK	*LMNA*	*RASA1*	*TTR*
DSC2	*LZTR1*	*RBM20*	*VCL*

(*) source: https://www.invitae.com/en/providers/test-catalog/test-02101, accessed 20220626

**Table S3.** Comprehensive Glycogen Storage Disease Panel*	
AGL	*PFKM*
ALDOA	*PGAM2*
ENO3	*PGK1*
FBP1	*PGM1*
G6PC	*PHKA1*
GAA	*PHKA2*
GBE1	*PHKB*
GYG1	*PHKG2*
GYS1	*POLG*
GYS2	*PYGL*
LAMP2	*PYGM*
LDHA	*RBCK1*
LPIN1	*SLC2A2*
PCK1	*SLC37A4*
PCK2	

(*) source: https://www.invitae.com/en/providers/test-catalog/test-06156, accessed 20220626
